# High antimicrobial resistance rates and multidrug resistance in *Enterobacteriaceae* isolates from poultry in Souk Ahras region, Algeria

**DOI:** 10.14202/vetworld.2024.2709-2718

**Published:** 2024-12-06

**Authors:** Khaoula Kamel, Amina Merghad, Djanette Barour, Djalel Eddine Gherissi, Tarek Khenenou

**Affiliations:** 1 Department of Veterinary Sciences, Institute of Agronomic and Veterinary Sciences, University of Mohamed Cherif Messaâdia, Souk Ahras, Algeria; 2 Laboratory of Science and Techniques for Living, Institute of Agronomic and Veterinary Sciences, University of Mohamed Cherif Messaâdia, Souk Ahras, Algeria; 3 Aquatic and Terrestrial Ecosystems Laboratory, Faculty of Nature and Life Sciences, University of Mohamed Cherif Messaâdia, Souk Ahras, Algeria; 4 Department of Biology, Faculty of Nature and Life Sciences: University of Mohamed Cherif Messaâdia, Souk Ahras, Algeria; 5 Laboratory of Animal Production, Biotechnology and Health, Institute of Agronomic and Veterinary Sciences, University of Mohamed Cherif Messaâdia, Souk Ahras, Algeria

**Keywords:** Algeria, antibiotic resistance, *Enterobacteriaceae*, multidrug resistance, poultry

## Abstract

**Background and Aim:**

The spread of antimicrobial resistance (AMR) in the *Enterobacteriaceae* family represents a major global health problem for humans and animals. This study aimed to determine AMR levels and highlight the different resistance profiles of *Enterobacteriaceae* isolates collected from healthy broiler chickens in eastern Algeria.

**Materials and Methods:**

A total of 200 cloacal swabs of healthy broilers from several poultry farms located in the Souk Ahras region (eastern Algeria) were collected. Samples were inoculated on MacConkey agar, and the isolated bacteria were identified using the API 20E system. Antimicrobial susceptibility testing was conducted using the disk diffusion method in accordance with the Clinical and Laboratory Standards Institute guidelines. The broth microdilution method was used to determine the minimum inhibitory concentration of colistin (CT).

**Results:**

Two hundred and forty-one isolates of commensal *Enterobacteriaceae* were recovered, including: *Escherichia coli* (n = 194; 80.5%), *Proteus mirabilis* (n = 21; 8.71%), *Escherichia fergusonii* (n = 8, 3.32%), *Salmonella* spp. (n = 7, 2.9%)*, Enterobacter cloacae* (n = 4, 1.66%), *Klebsiella pneumoniae* (n = 3, 1.25%), *Serratia* spp. (n = 3, 1.25%), and *Kluyvera* spp. (n = 1, 0.41%). High resistance rates were observed toward erythromycin (100%), doxycycline (96.68%), trimethoprim-sulfamethoxazole (95.85%), ciprofloxacin (94.19%), ampicillin (90.04%), kanamycin (78.01%), and amoxicillin-clavulanic acid (69.71%). However, moderate-to-low resistance rates were observed for CT (25.31%), ceftazidime (12.45%), and cefotaxime (8.71%). Interestingly, only two extended-spectrum beta-lactamase (ESBL)-producing *E. coli* isolates were detected. All isolates (100%) were multidrug-resistant (MDR), among which 58.92% were resistant to six and seven antibiotics. Forty AMR profiles were identified, reflecting a wide diversity of resistance with combinations of three to ten antibiotics.

**Conclusion:**

Our findings revealed alarming rates of AMR, highlighting the need to take measures to combat the phenomenon of AMR to protect animals and public health.

## Introduction

Globally, antimicrobial resistance (AMR) has emerged as a real threat to human and animal health. AMR was ranked as one of the top ten global health threats by the World Health Organization (WHO) in 2014; this ranking has not changed in nearly a decade [1]. The rapid spread of antimicrobial-resistant bacteria (ARB) and antimicrobial resistance encoding genes (ARGs) between humans, animals (livestock and wildlife), plants, and their shared environment [2] has required the use of the global “One Health” approach to combat the AMR issue [3]. The interconnectivity of the aforementioned ecological niches leads to the spread of ARBs and ARGs through numerous pathways, including direct contact between humans and animals, the food chain, environmental contamination by human and animal waste, and the movement of wildlife, particularly wild migratory birds [2, 4].

AMR has long been a concern in livestock, leading to numerous therapeutic failures. It also represents a public health risk because ARBs and ARGs can be transferred to humans. Indeed, farmed food animals at an industrial scale and their surrounding environment are recognized as hot spots for ARBs and ARGs due to the common use of antimicrobials for prophylactic purposes as well their heavy use as growth promoters, despite their banning by several countries [1]. The heavy use of antimicrobial agents for therapeutic purposes against pathogenic bacteria or as growth promoters exert selective pressure not only on infectious bacteria but also on commensal ones such as those belonging to *Enterobacteriaceae* [2]. Consequently, animal-derived ARBs have shown a clonal relationship with human bacteria, indicating their likelihood of transfer to humans mainly through the food chain [3]. The genetic relationships between human and poultry-derived strains of zoonotic bacteria, such *as Escherichia coli*, have been particularly noted. This observation is consistent with the recognition that industrial poultry farming is a major consumer of antimicrobial agents and can act as a source and reservoir for ARBs and ARGs [4, 5].

Infections caused by *Enterobacteriaceae* in poultry, especially colibacillosis caused by pathogenic *E. coli*, are a well-known global problem, causing significant economic losses in the poultry sector and representing one of the main reasons for seizures during slaughterhouse inspections [6]. Commonly, beta-lactams and fluoroquinolones are used to treat these infections; however, infections caused by strains that are resistant to these antibiotics or by multidrug-resistant strains limit the number of available treatment options and increase the severity of infections [7]. Such resistant/MDR strains can reach humans, especially through the food chain or by contamination of terrestrial and aquatic environments, which poses a serious risk to public health and food safety.

Benklaouz *et al*. [8], Benameur *et al*. [9], Barour *et al*. [10], Belmahdi *et al*. [11] have reported high rates of AMR in *E. coli* isolates of avian origin in Algeria. However, data on the AMR of commensal *Enterobacteriaceae* species other than *E. coli* are scarce in poultry farms. Therefore, in this study, we aimed to investigate AMR in *Enterobacteriaceae* isolates from healthy broiler chickens, highlight the various resistance profiles, and assess the multidrug resistance level of collected isolates in the Souk Ahras region, East Algeria.

## Materials and Methods

### Ethical approval

The sampling protocol did not require any ethical approval from the ‘University Animal Ethics Committee’ and was performed in accordance with Algerian laws and regulations on animal welfare.

### Study period and location

This study was conducted from May 2022 to June 2023. The samples were collected from several poultry farms in the Souk Ahras region in eastern Algeria. The samples were processed at the Laboratory of Science and Techniques for Living, Institute of Agronomic and Veterinary Sciences, University of Souk Ahras, Algeria.

### Sampling

This study used the following formula for the sample size calculation: n=Z^2^.p. (1-p)/E^2^

Where.

n = required sample size,

Z = z-value associated with the desired confidence level (1.96 for 95 % confidence),

p = estimated population proportion,

E = the allowable error margin at 5%

For this study, P was taken as 56% based on previous findings by Benameur *et al*. [9]. By using this formula, we derived a sample size of 378. Since it was impossible to collect this number of samples and to study a high number of *E. coli* isolates owing to technical and financial limitations, for these reasons and according to several studies [8–10], we judged that 200 samples would be suitable for this study. Therefore, two hundred cloacal swabs of apparently healthy broiler chickens were collected using sterile swabs. The samples were stored at 4°C and transferred to the laboratory for bacteriological analysis within 24 h.

### Isolation and identification

Before isolation, an enrichment step was performed by introducing each swab loaded with fecal material into 9 mL of buffered peptone water (Pasteur Institute of Algeria), followed by overnight incubation at 37°C. Then, bacterial isolation was performed by streaking enriched samples on MacConkey agar (Merck, Darmstadt, Germany) for 24 h at 37°C. One or two colonies were selected and re-isolated from each sample on MacConkey agar to obtain pure colonies. Each pure bacterial isolate was subjected to preliminary identification using classical biochemical methods such as oxidase, catalase, urease testing, indole production, lactose oxidation, and glucose fermentation by Kligler-Hajna agar (Bio-Rad, Marnes-la-Coquette, France) The identification was confirmed by the API 20E system (BioMérieux, Marcy l’Etoile, France).

### Antimicrobial susceptibility testing

Antimicrobial susceptibility testing for all identified isolates was performed using the disk diffusion method on Mueller–Hinton agar (Merck). The results were interpreted according to the Clinical and Laboratory Standards Institute (CLSI) guidelines [12]. The following antibiotics (Oxoid Ltd, England) were tested: Ampicillin (AMP, 10 μg), cefotaxime (CTX, 30 μg), ceftazidime (CAZ, 30 μg), amoxicillin/clavulanic acid (AMC, 20/10 μg), ciprofloxacin (CIP, 5 μg), kanamycin (K, 30 μg), trimethoprim-sulfamethoxazole (SXT, 1.25/23.75 μg), doxycycline (DO, 30 μg), and erythromycin (E, 15 μg).

For colistin (CT) susceptibility, the minimum inhibitory concentration (MIC) was determined using the broth microdilution technique in Mueller–Hinton broth following the CLSI guidelines [12]. The Double-Disk Synergy Test (DDST) was used to detect extended-spectrum beta-lactamase (ESBL) production by placing each of the CTX (30 μg) and ceftazidime (30 μg) disks at a distance of 3 cm from the central disk of amoxicillin-clavulanic acid (20/10 μg) [12]. *E. coli* ATCC 25922 and *Klebsiella pneumoniae* ATCC 700603 were used as ESBL-negative and positive reference strains, respectively.

### Statistical analysis

Microsoft Office Excel 2007 (Microsoft Office, Washington, USA) was used to create the graphic representation. The statistical approach elucidated the complex interplay between antibiotic resistance and bacterial species within the *Enterobacteriaceae* population. For this purpose, R statistical software (R Foundation for Statistical Computing, Vienna, Austria. Version 4.3.3) was used. To further explore the relationship between antibiotic resistance and bacterial species, contingency tables were constructed, and the Chi-square test was applied to determine the significance of differences in resistance rates. Furthermore, a dendrogram of resistance profiles within *Enterobacteriaceae* isolates was generated to visually classify clusters based on Euclidean distance. This dendrogram allowed the identification of groups exhibiting similar antibiotic resistance patterns. Significance levels were set at 0.05, 0.01, and 0.001, providing thresholds for determining the statistical significance of the observed results.

## Results

### Isolation of *Enterobacteriaceae*

**Figure-1:**
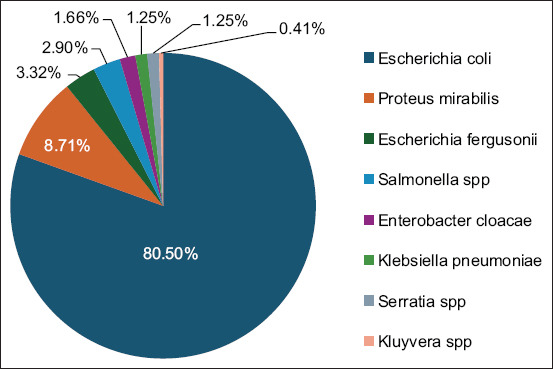
Frequencies of the identified *Enterobacteriaceae* species.

Two hundred and forty-one *Enterobacteriaceae* isolates were recovered from 200 cloacal swabs collected from various broiler chicken farms in the Souk Ahras region. *E. coli* was the most isolated bacterium (n = 194, 80.50%), followed by *Proteus mirabilis* (n = 21, 8.71%), *Escherichia fergusonii* (n = 8, 3.32%), *Salmonella* spp. (n = 7, 2.90%), *Enterobacter cloacae* (n = 4, 1.66%), *K. pneumoniae* (n = 3, 1.25%), *Serratia* spp. (n = 3, 1.25%), and *Kluyvera* spp. (1, 0.41%) (Figure-1).

### Antimicrobial susceptibility

**Figure-2:**
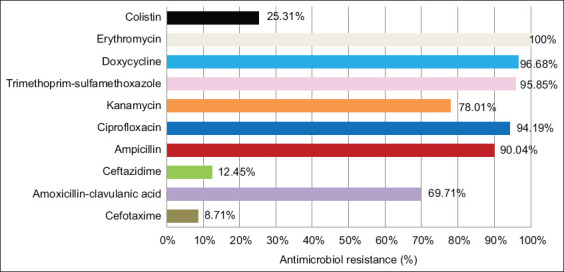
The percentage of antimicrobial resistance in 241 *Enterobacteriaceae* isolates.

**Figure-3:**
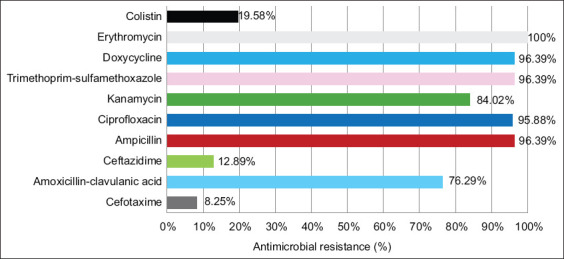
Percentage of antibiotic resistance among 194 *Escherichia coli* isolates.

Overall, the 241 isolates showed high rates of AMR against the majority of tested antibiotics (Table-1 and Figure-2): E (n = 241, 100%), DO (n = 233, 96.68%), SXT (n = 231, 95.85%), CIP (n = 227, 94.19%), AMP (n = 217, 90.04%), K (n = 188, 78.01%), and AMC (n = 168, 69.71%). However, low rates were observed for CAZ (n = 30, 12.45%) and CTX (n = 21, 8.71%). Interestingly, CT resistance was observed in 61 (25.31%) out of the 241 isolates. Interestingly, these rates were closely similar to those observed in the 194 *E. coli* isolates (Figure-3): E (100%), DO (96.39%), SXT (96.39%), CIP (95.88%), AMP (96.39%), K (84.02%), AMC (76.29%), CAZ (12.89%), CTX (8.25%), and CT (19.58%) (Table-1 and Figure-3). Notably, among the 241 isolates, only two *E. coli* isolates were ESBL producers, as indicated by the DDST. For the other species, the resistance rates are presented in Table-1.

**Table-1:** Antibiotic resistance in isolated *Enterobacteriaceae* species.

Isolates (N)	Tested antibiotics N (%)										P1-value

	Cefotaxime	Amoxicillin- clavulanic acid	Ceftazidime	Ampicillin	Ciprofloxacin	Kanamycin	Trimethoprim- sulfamethoxazole	Doxycycline	Erythromycin	Colistin	
*Escherichia coli* (194)	16 (8.25)	148 (76.29)	25 (12.89)	187 (96.39)	186 (95.88)	163 (84.02)	187 (96.39)	187 (96.39)	194 (100)	38 (19.58)	***
*Proteus mirabilis* (21)	0 (0)	3 (14.29)	0 (0)	6 (28.57)	18 (85.71)	11 (52.38)	20 (95.24)	21 (100)	21 (100)	12 (57.14)	***
*Escherichia fergusonii* (8)	5 (62.5)	5 (62.5)	4 (50)	7 (87.50)	6 (75)	5 (62.50)	6 (75)	8 (100)	8 (100)	4 (50)	ns
*Salmonella* spp. (7)	0 (0)	5 (71.43)	1 (14.29)	6 (85.71)	7 (100)	5 (71.43)	7 (100)	7 (100)	7 (100)	3 (42.86)	***
*Enterobacter cloacae* (4)	0 (0)	3 (75)	0 (00)	4 (100)	3 (75)	2 (50)	4 (100)	4 (100)	4 (100)	1 (25)	**
*Klebsiella pneumoniae* (3)	0 (0)	1 (33.33)	0 (00)	3 (100)	3 (100)	1 (33.33)	3 (100)	3 (100)	3 (100)	1 (33.33)	*
*Serratia* spp. (3)	0 (0)	2 (66.67)	0 (0)	3 (100)	3 (100)	0 (0)	3 (100)	2 (66.67)	3 (100)	1 (33.33)	**
*Kluyvera* spp. (1)	0 (0)	1 (100)	0 (0)	1 (100)	1 (100)	1 (100)	1 (100)	1 (100)	1 (100)	1 (100)	ns
Total (241)	21 (8.71)	168 (69.71)	30 (12.45)	217 (90.04)	227 (94.19)	188 (78.01)	231 (95.85)	233 (96.68)	241 (100)	61 (25.31)	/
P2-value	***	***	*	***	***	***	ns	ns	ns	**	/

N: Number of isolates,

* p < 0.05,

** p < 0.01,

*** p < 0.001, ns=not significant (The level of statistical significance was set at P < 0.05). P1: The value represents the significance of differences in resistance rates among tested antibiotics for each bacterial species. P2: The value represents the significance of differences in resistance rates among bacterial species for each tested antibiotic

When comparing the resistance rates of each isolated *Enterobacteriaceae* species to tested antibiotics, a significant difference (p < 0.05) in antibiotic resistance rates among all studied *Enterobacteriaceae* isolates was observed, except for DO, SXT, and E (p > 0.05). In addition, when comparing the resistance rates of each isolated *Enterobacteriaceae* species to the antibiotics tested, a significant difference in resistance rates (p < 0.05, p < 0.01, and p < 0.001) was observed for the isolates of *E. coli, P. mirabilis*, *Salmonella* spp., *E. cloacae*, *K. pneumoniae*, and *Serratia* spp., isolates (Table-1). In this regard, it is worth noting that *E. coli* isolates exhibited high resistance rates toward AMC (76.29%), AMP (96.39%), CIP (95.88%), K (84.02%), SXT (96.39%), DO (96.39%), and E (100%). However, *P. mirabilis* showed low resistance rates (0%–28.57%) to AMC, AMP, CTX, and CAZ, but high resistance rates to the remaining antibiotics (52.38%–100%).

Regarding *E. fergusonii*, resistance to CAZ and CT was moderate (50%), whereas resistance to other antibiotics (62.50%–100%). For *Salmonella* spp. isolates, none were CTX resistant; however, 14.29% were CAZ -resistant, and high rates were observed for the remaining antibiotics (42.86%–100%). Among the four *E. cloacae* isolates, none was CTX resistant, CT resistance was detected in two isolates (25%), and high resistance rates were detected for the other antibiotics (75%–100%). CTX and CAZ resistance was not detected in the three *K. pneumoniae* isolates; however, CT resistance was observed in one isolate, and some isolates were resistant to other tested antibiotics (Table-1). In addition, for the three *Serratia* spp. isolates, no resistance or low rates were observed for CTX, CAZ, K, and CT (0%–33%). Finally, the unique *Kluyvera* spp. identified were pan-resistant to all tested antibiotics except CTX and CAZ (Table-1).

### Co-resistances and MDR rates

**Figure-4:**
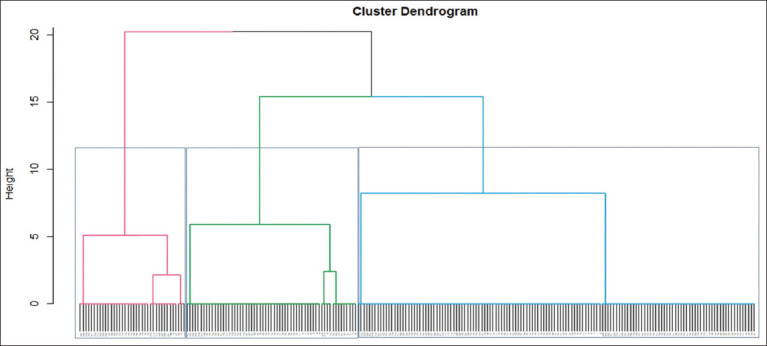
Dendrogram illustrating clusters of multidrug resistance among 241 *Enterobacteriaceae* isolates. The bottom margin indicates the isolates in each cluster. The left margin indicates the Euclidean distance between different clusters.

Analysis of the AMR pattern of each isolate among the 241 isolates showed that all of them were resistant to three to ten antimicrobial agents, which defined them as multidrug-resistant (MDR) (Table-2 and Figure-4). Indeed, 40 resistance profiles were observed among the 241 isolates, and according to Table-2 and Figure-4, these isolates were subdivided into three clusters. Cluster 1 contained 142 (58.92%) of the total isolates, followed by cluster 2 and cluster 3 containing 61 (25.31%) and 38 (15.77%) isolates, respectively. Cluster 1 contained isolates demonstrating resistance to 6 and 7 antibiotics. Similarly, cluster-2 encompassed isolates exhibiting resistance to 8, 9, and 10 antimicrobials. Finally, cluster 3 contained isolates resistant to 3, 4, and 5 antibiotics (Table-2 and Figure-4). The most common resistance profile (P25), observed in 67 (27.80%) isolates, corresponds to resistance against seven types of antimicrobials, including AMP, AMC, CIP, K, DO, SXT, and E. In addition, P21 (AMP, AMC, CIP, K, DO, SXT, and E) and P35 (AMP, AMC, CIP, K, DO, SXT, E, and CT) were detected in 28 (11.62%) isolates (Table-2). The remaining resistance patterns were identified for 1–18 isolates.

**Table-2:** Patterns of antibiotic resistance phenotypes of 241 *Enterobacteriaceae* isolates (40 patterns).

Clusters N (%)	The number of resistances	Resistance patterns	Number of resistant isolates N (%)
Cluster 3. 38 (15.77)	3	P1: CIP, SXT, and E.	1 (0.41)
		P2: AMP, DO, E, and	1 (0.41)
		P3: CIP, E, and DO	1 (0.41)
	4	P4: AMP, CIP, SXT, and E	2 (0.83)
		P5: AMP, DO, SXT, and E	1 (0.41)
		P6: CIP, SXT, DO, E, and	4 (1.66)
		P7: K, SXT, DO, E.	1 (0.41)
		P8: SXT, DO, E, CT	2 (0.83)
	5	P9: AMP, AMC, CIP, DO, E, and	2 (0.83)
		P10: AMP, CIP, DO, SXT, and E.	10 (4.15)
		P11: AMP, K, DO, SXT, and E.	1 (0.41)
		P12: AMP, CIP, K, AMC, E, and	1 (0.41)
		P13: AMC, K, DO, SXT, and E.	1 (0.41)
		P14: CIP, K, DO, E, and SXT.	2 (0.83)
		P15: CIP, SXT, DO, E, and CT	3 (1.24)
		P16: AMP, AMC, CIP, SXT, and E	1 (0.41)
		P17: AMC, CIP, DO, SXT, and E.	3 (1.24)
Cluster 1. 142 (58.92)	6	P18: AMP, AMC, K, DO, SXT, and E.	3 (1.24)
		P19: AMC, DO, SXT, E, CIP, and K	1 (0.41)
		P20: AMP, AMC, CIP, DO, SXT, and E	18 (7.47)
		**P21: AMP, CIP, K, DO, SXT, and E.**	**28 (11.62)**
		P22: AMP, AMC, CIP, DO, E, and K	1 (0.41)
		P23: CIP, K, SXT, DO, E, and CT	4 (1.66)
		P24: AMP, AMC, CIP, SXT, E, and CT	1 (0.41)
	7	**P25: AMP, AMC, CIP, K, DO, SXT, and E**	**67 (27.80)**
		P26: AMP, CIP, K, DO, SXT, E, and CT	11 (4.56)
		P27: AMC, AMP, CTX, CIP, DO, E, and K	2 (0.83)
		P28: AMP, AMC, CIP, DO, SXT, E, and CT	2 (0.83)
		P29: AMP, AMC, K, DO, SXT, E, and CAZ	1 (0.41)
		P30: AMC, AMP, CAZ, CIP, SXT, DO	2 (0.83)
		P31: AMC, CTX, CAZ, K, DO, E, CT	1 (0.41)
		P32: AMP, AMC, K, DO, SXT, E, and CT	1 (0.41)
Cluster 2. 61 (25.31)	8	P33: AMP, AMC, K, DO, SXT, E, CIP, and CAZ	13 (5.39)
		P34: AMC, AMP, CIP, SXT, DO, E, CTX, and K	6 (2.49)
		**P35: AMP, AMC, CIP, K, DO, E, SXT, CT**	**28 (11.62)**
		P36: AMC, AMP, CTX, CAZ, K, DO, E, and CT	1 (0.41)
	9	P37: AMP, AMC, CTX, CAZ, CIP, K, DO, SXT, and E	6 (2.49)
		P38: AMP, AMC, CIP, K, DO, SXT, E, CT, and CAZ	2 (0.83)
		P39: AMP, AMC, CTX, K, DO, SXT, E, CIP, CT	1 (0.41)
	10	P40: CTX, CAZ, AMC, AMP, CIP, K, DO, E, SXT, CT	4 (1.66)

AMP: Ampicillin, AMC: Amoxicillin-clavulanic acid, CTX: Cefotaxime, CAZ: Ceftazidime, CIP: Ciprofloxacin, K: Kanamycin, SXT: Trimethoprim-sulfamethoxazole, DO: Doxycycline, E: Erythromycin, CT: Colistin, N: Number of isolates. The resistance patterns in bold correspond to the most common patterns. P1 to P40: Resistance patterns

## Discussion

The present study aimed to investigate the AMR phenomenon in *Enterobacteriaceae* isolates from healthy broiler chickens and to investigate the risk of AMR of *Enterobacteriaceae* colonizing healthy broiler chickens in eastern Algeria. *Enterobacteriaceae* is widely found in the natural environment and in the digestive systems of birds, mammals, and humans. Several genera and species belonging to this family are major pathogenic bacteria in human clinical settings, as well as in food-producing animals; however, *Kluyvera* genus is scarcely related to clinical infection. Enterobacteria pathogens include *E. coli*, *K. pneumoniae*, *Klebsiella* *oxytoca*, *E. cloacae*, *Proteus* spp., *Citrobacter* spp., *Serratia marcescens*, and *Salmonella* spp. [13, 14]. Globally, AMR is increasing in several species of *Enterobacteriaceae*, particularly against beta-lactams, quinolones, fluoroquinolones, aminoglycosides, and CT [15]. Due to their potential to spread to humans as foodborne pathogens, ARB in animals poses a growing threat. Because *Enterobacteriaceae* species are among the most significant and abundant human pathogens, their antibiotic susceptibility must be monitored.

From 200 cloacal swabs collected from apparently healthy broiler chickens, 241 *Enterobacteriaceae* isolates were recovered and studied. *E*. *coli* was the most isolated bacterium (80.50%), followed by *P. mirabilis* (8.71%), *E. fergusonii* (3.32%), *Salmonella* spp. (2.90%)*, E. cloacae* (1.66%)*, K. pneumoniae* (1.25%), *Serratia* spp. (1.25%), and *Kluyvera* spp. (0.41%). The occurrence of some of these species has been previously reported in avian farms in Algerian by Benklaouz [16], where 315 *Enterobacteriaceae* strains were isolated with a predominance of *E. coli* (55.23%) followed by *Proteus* spp. (16.82%) isolates; in addition, a few isolates were identified as *Salmonella* spp. (1.90%). In another study conducted in Nigeria [17], involving 287 *Enterobacteriaceae* isolates collected from laying chicken, *E. coli* remained the most frequent (59.6%), but significant rates were also detected for *Enterobacter* spp. (27.9%) and *K. pneumoniae* (12.5%). The predominance of *E. coli* is not surprising; indeed, it is the most prevalent enteric bacterium in the gastrointestinal tract of poultry [18, 19].

The *Enterobacteriaceae* isolates showed very high resistance to E (100%), DO (96.68%), SXT (95.85%), CIP (94.19%), AMP (90.04%), K (78.01%), and AMC (69.71%). Moderate resistance was observed for CT (25.31%) and CAZ (12.45%), whereas low resistance was noted for CTX (8.71%). Interestingly, only two ESBL-producing *E. coli* isolates were detected.

The high rate of E resistance, similar to that reported previously by Kilonzo-Nthenge *et al*. [20], might be explained in part by the heavy use of E in the studied farms; however, no data are available about its use in the studied farms, but its use is a common practice in avian husbandry in Algeria and other countries. E has been widely used in fish, poultry, and livestock as a growth promoter and to prevent bacterial infections. In poultry production, E is mainly incorporated in feed to prevent chronic respiratory diseases during periods of stress, to prevent infectious coryza caused by *Avibacterium* *paragallinarum*, and to prevent and reduce lesions [21, 22]. Globally, the noteworthy result of excessive E use in avian husbandry is the global increase in E-resistant *Campylobacter* spp. of poultry and turkey origin.

An alarming rate of resistance was also noted for DO (98.68%), a tetracycline antibiotic, which was also noted. This finding is similar to that reported in Pakistan [23], where the resistance rate was 84.42%. Globally, tetracyclines have become the most frequently used antibiotics in livestock and poultry farming owing to their numerous advantages, including low cost, high efficiency, and wide range of action [24]. Since the discovery of tetracyclines in the 1940s, they have been used for the treatment of infectious diseases and growth promotion. However, these broad applications have led to the rapid emergence of bacterial strains resistant to this antimicrobial family [25, 26].

SXT combination is the broad-spectrum antibiotics used in poultry for the prophylaxis and treatment of bacterial infections caused by *E. coli*, *Haemophilus*, and *Pasteurella* spp. [27]. However, prolonged and uncontrolled use of these antimicrobials (trimethoprim and sulfonamides) in animal feed and human medicine can lead to the development and transmission of genes encoding these resistance markers [28]. In our isolates, a high rate of SXT resistance (95.85%) was detected. These results are consistent with those reported by He *et al*. [29]. In contrast, Yulistiani *et al*. [30] reported a lower resistance rate of 42.36%. Resistance to trimethoprim and sulfonamide is mainly encoded by genes localized on mobile genetic elements such as plasmids and class 1 integrons, which enhance their large spread within and between bacterial pathogens [28].

Beta-lactams are the most commonly employed antibiotics for treating infections caused by *Enterobacteriaceae*. Resistance to these antibiotics arises through mutations or the acquisition of exogenous genetic materials, such as plasmids, transposons, and integrons, from other resistant bacteria [31]. Beta-lactam antibiotic class is extensively used in eastern Algeria [32, 33]. Our results indicate a high rate of AMP resistance (90.04%), similar to those reported in Egypt [34], Mozambique [35] and Ethiopia [36]. Our results indicate a high rate of AMC resistance (69.71%), which is similar to the findings reported by Kamboh *et al*. [23] and Bushen *et al*. [36].

Cephalosporins are considered first-line antibiotics for treating bacterial infections in humans [37]. The results of our study revealed a relatively low resistance rate for third-generation cephalosporins (8.71% for CTX and 12.45% for CAZ), which can be attributed to the rare use of these molecules (C3G) in poultry farms in Algeria [38]. Our results are lower than those reported by Kamboh *et al*. [23], who reported rates of (41.13%) for CTX and (33.33%) for CAZ. Faife *et al*. [35] observed higher resistance rates for CTX (74%) and CAZ (67%). Interestingly, in this study, only two ESBL-producing *E. coli* isolates were identified using the DDST assay. Our results concerning ESBL-producing strains are similar to those previously reported by Barour *et al*. [10] and Aberkane *et al*. [39] in Algeria and lower than those reported by Benklaouz [16] in Algeria and Cardozo *et al*. [40] in Brazil.

Genes encoding ESBLs in *Enterobacteriaceae* are frequently located on plasmids that also carry genes for resistance to other classes of antibiotics, such as fluoroquinolones and aminoglycosides [41]. Globally, poultry has the highest prevalence of ESBL-producing *E. coli*, with the spread of ESBL genes through both vertical cell division and genetic transfer within the species. In addition, this spread occurs horizontally to other species and genera through plasmid conjugation or the transposition process [42–44]. Notably, most international studies reporting high rates of ESBL producer isolates from poultry samples used selective medium (containing 2–4 mg/CTX) [11, 40]. However, this strategy was not adopted in our study, and random colonies were further selected and studied, which explains the low rate of ESBL producer isolates.

For fluoroquinolones (CIP), a very high level of resistance has been reported, which is consistent with the results of Benameur *et al*. [45] in western Algeria. However, these values are higher than those found in the previous studies, such as in China [29], Mozambique [35], and Ethiopia [46]. The high resistance rate of *Enterobacteriaceae* to quinolones/fluoroquinolones in poultry in Algeria is of concern, probably due to the administration of these antibiotics for prophylactic and therapeutic purposes and their use for the prevention of early mortality in chicks [47]. Globally, the rise of quinolone/fluoroquinolone resistance has been associated with ESBL and carbapenemase production due to the co-occurrence of their genetic determinants on the same mobile genetic elements [29, 35, 40, 46]. Our study observed a high resistance rate (78.01%) for K, which is higher than reported by Kilonzo-Nthenge *et al*. (17.90%) [20].

WHO [48] classified CT, also known as polymyxin E, as the highest priority critically important antibiotic. Indeed, CT is one of the last-resort antibiotics used to treat MDR and ESBL- or carbapenemase-producing Gram-negative bacilli [49]. In the studied isolates, a relatively moderate CT resistance rate (25.31%) (MICs ≥4 μg/mL) was detected. In contrast, Merazi *et al*. [50] reported a high rate of CT resistance (48.98%) from enterobacteria isolates collected from sick and dead broilers. In another study from Iran, CT resistance was detected only in 9 (0.96%) *K. pneumoniae* isolates among 931 enteric isolates collected from healthy and sick chickens and turkeys [51]. Similarly, a low resistance rate to CT was also observed in Malaysia, where 7.2% of avian *Enterobacteriaceae* isolates exhibited phenotypic CT resistance [52]. In summary, the rates of CT resistance appear to vary geographically, which may be related to differences in CT use at the farm level.

The analysis of the AMR patterns of all isolates showed that all isolates were MDR and exhibited resistance to at least three or more antimicrobial agents. This finding is similar to those reported in previous studies by Benklaouz [16] and Benameur *et al*. [45]. This increase in multidrug resistance rates can be explained by the widespread use of antimicrobials for therapeutic purposes or growth promotion in poultry farms [10]. The most common resistance pattern was the combination of resistance to AMP, AMC, CIP, K, DO, SXT, and E; all belong to six antimicrobial families. The phenotypic MDR trait is mainly the result of genetic co-localization of various ARGs conferring different resistance phenotypes on mobile genetic elements such as plasmids, transposons, and integrons [53]. These genetic elements cause a high risk of spreading AMR through horizontal gene transfer. Furthermore, on acquisition, these mobile genetic elements could also integrate other resident ARGs of host bacteria. This complex process of genetic combinations leads to the formation of mosaic mobile genetic elements containing multiple ARGs.

Our study highlighted high AMR rates and MDR phenotypes in *Enterobacteriaceae* isolates from healthy poultry in Algeria. The spread of such strains to terrestrial and aquatic environments, as well as to other food-producing animals and humans through various pathways is a cause of concern to public health. This likelihood scenario ultimately confirms the need for the “One Health” approach in Algeria to fight the emergence and spread of ARBs and ARGs. Since October 2020, Algeria has been a member of the Global AMR and Use Surveillance System (GLASS), the first global collaborative effort to standardize AMR surveillance (https://www.who.int/initiatives/glass). Since that date, the Algerian Antibiotic Resistance Network (AARN) (http;//aarn.pasteur.dz), a member of the GLASS, has continued to provide annual reports about AMR in several Algerian hospitals as well as to organize “Seminar Workshops” for a wide range of healthcare participants. Unfortunately, such surveillance systems have not yet been established in veterinary medicine. However, significant research and academic studies have been performed on food-producing animals and wildlife in Algeria, which will certainly lead to the establishment of a veterinary AMR surveillance system that reinforces the AARN.

The limitations of this study are the lack of molecular investigation of genes encoding AMR and the genetic support of those genes that would be studied by determining the occurrence of integrons and the plasmid types (incompatibility groups) known as vehicles of AMR genes and virulence genes. In addition, investigation of the clonal relationship of collected isolates, especially the dominant species *E. coli*, using molecular methods such as multilocus sequence typing will be useful to better understand the genetic structure of these bacterial populations and to gain insight into the molecular mechanisms of AMR within and between farms and zoonotic avian bacteria.

## Conclusion

Our findings revealed that *Enterobacteriaceae* isolates from broilers in the Souk Ahras region have a high resistance level against most antibiotics tested (E, DO, SXT, CIP, AMP, K, and AMC). In addition, two strains of *E. coli* producing ESBL were detected. The majority of isolates exhibited MDR phenotype. Indeed, forty resistance profiles with combinations of three to ten different antibiotics were identified. The high AMR levels observed in bacterial isolates from poultry farms could have significant implications for human and animal health; thus, urgent measures are required to combat and reduce AMR in the poultry sector.

## Authors’ Contributions

AM and DB: Conceived and designed the study. KK: Collected and analyzed the data and performed the experiments. KK and DB: Analyzed the data and drafted the manuscript. DEG: Statistical analysis. AM and TK: Interpreted the data and revised and finalized the manuscript. All authors have read and approved the final manuscript.
